# True unicornuate uterus- Pseudounicornuate uterus

**Published:** 2012-07

**Authors:** Firoozeh Ahmadi, Hadieh Haghighi

**Affiliations:** *Department of Reproductive Imaging, Reproductive Biomedicine Research Center, Royan Institute for Reproductive Biomedicine, ACECR, Tehran, Iran.*

**Figure 1:**
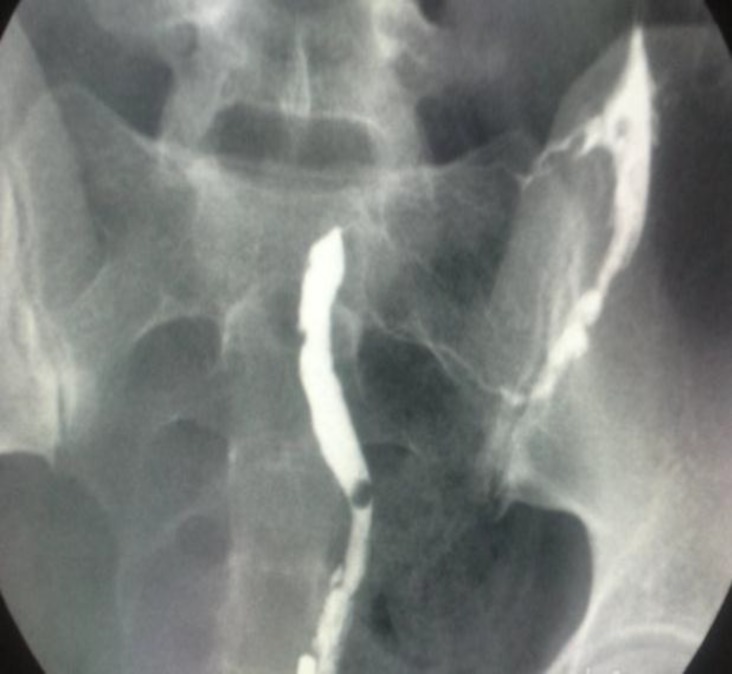
Later HSG demonstrates apparently unicornuate uterus with normal left fallopian tube

**Figure 2:**
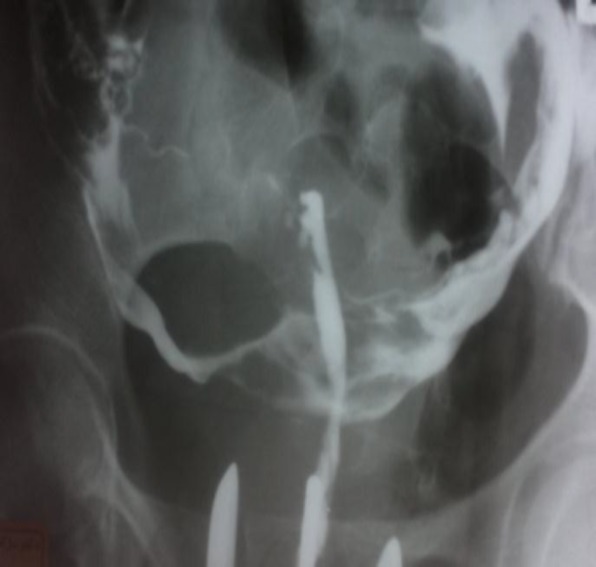
First hysterosalpingography (HSG) showed a apparently unicornuate uterus with normal fallopian tubes

A 27 year old patient presented with primary infertility of 3 years' duration and also a history of myomectomy (5 years ago) was referred to our infertility clinic for investigation of infertility. The latest Hysterosalpingography (HSG) revealed an obstructed left fallopian tube with apparently a unicornuate uterus with luminal contour irregularity and normal left fallopian tube (Figure 1). Significant information in her past medical history revealed that she had another HSG two years before and her first hysterosalpingography (HSG) showed a apparently unicornuate uterus. Additional significant information in comparison with second HSG revealed that both fallopian tubes were opacified (Figure 2). In this case medical history also included hysteroscopic diagnosis of adhesion following open myomectomy at the age of 22. Comparison of previous graphies and hysteroscpic findings lead to a suggestion of pseudounicornuate uterus. Intrauterine adhesions develop after trauma to the basal layer of the endometrium. Unilateral excessive scarring of the uterus may lead to an obliteration of the uterine lumen resulting in an image that can mimic a unicornuate uterus (pseudounicornuate uterus) (1). A true unicornuate uterus should be excluded from pseudounicornuate uterus by a) horizontally oriented in its long axis due to deficient development of mullerian ducts b) smooth or regular contour c) with one tube. While pseudounicornuate uterus look like acquired lesion and cicatrisation leads to a usually irregular contour and uterus is more vertical in its long axis (2). Obtaining an accurate history, comparison of previous sonographic or laparoscopic findings, and awareness about this image of synechiae are the critical steps in differentiating a pseudounicornuate uterus from true unicornuate uterus.
